# Effects of thyroid hormones on major cardiovascular risk in acute coronary syndromes

**DOI:** 10.1186/cc9421

**Published:** 2011-03-11

**Authors:** A Bayrak, A Bayır, K Uçar Karabulut

**Affiliations:** 1 Selçuk University, Meram Faculty of Medicine, Konya, Turkey; 2 Selçuk University, Selçuklu Faculty of Medicine, Emergency Department, Konya, Turkey; 3 Emergency Sercice of Şırnak State Hospital, Şırnak, Turkey

## Introduction

In this study we aimed to investigate the relationship between thyroid hormone abnormalities and major cardiovascular events and sudden cardiac death at 3 and 6 months after discharge in patients who were admitted to the Emergency Department with acute coronary syndrome.

## Methods

The study group included 110 patients without known thyroid dysfunction who were referred to the Emergency Department with acute coronary syndrome. FT3, FT4 and TSH levels were measured in all patients on admission. Patients were divided into STEMI, NSTEMI and UAP groups. Patient records were checked at 3 and 6 months of discharge in terms of sudden cardiac death and major cardiovascular events. The relationship between thyroid hormone levels and acute cardiac death and major cardiovascular disorders at 3 and 6 months of discharge was evaluated.

## Results

The mean TSH, FT3 and FT4 levels of the study group versus control group were as follows: TSH levels of study group 1.87 ± 1.73 μIU/ml, FT3 3.2 ± 1.34 pg/ml, FT4 1.45 ± 0.64 ng/dl. Abnormalities in the thyroid function tests were noted in 26 patients (23.6%). Of these seven patients (6.36%) had subclinical hypothyroidism, two patients (1.8%) had euthyroid sick syndrome and 10 patients (9%) had high serum FT4 levels despite normal FT3 and TSH values.

## Conclusions

We noted subclinical hypothyroidism, less frequently euthyroid sick syndrome and hyperthyroidism. No relationship was noted between thyroid hormone levels and sudden cardiac death and major cardiovascular disorders at 3 and 6 months follow-up. However, studies including larger patient groups are needed to clarify if there is a relationship between thyroid hormone levels on admission and sudden death and major cardiovascular events in patients with acute coronary syndrome.
